# Dose-Response Effects of the Text4baby Mobile Health Program: Randomized Controlled Trial

**DOI:** 10.2196/mhealth.3909

**Published:** 2015-01-28

**Authors:** William Evans, Peter E Nielsen, Daniel R Szekely, Jasmine W Bihm, Elizabeth A Murray, Jeremy Snider, Lorien C Abroms

**Affiliations:** ^1^Milken Institute School of Public HealthDepartment of Prevention and Community HealthThe George Washington UniversityWashington, DCUnited States; ^2^General Leonard Wood Army Community HospitalDepartment of Obstetrics and GynecologyFort Leonard Wood, MOUnited States; ^3^Madigan Army Medical CenterDepartment of Obstetrics and GynecologyTacoma, WAUnited States; ^4^School of Public HealthUniversity of WashingtonSeattle, WAUnited States

**Keywords:** mobile health, prenatal health care, health communication

## Abstract

**Background:**

Mobile health (mHealth) is growing rapidly, but more studies are needed on how to optimize programs, including optimal timing of messaging, dose of exposure, and value of interactive features. This study evaluates final outcomes of text4baby (a text message service for pregnant and postpartum women) from a randomized trial performed in a population of pregnant female soldiers and family members.

**Objective:**

The study aims were to evaluate (1) treatment effects and (2) dose-response effects of text4baby on behavioral outcomes compared to control (no text4baby) condition.

**Methods:**

The study was a randomized trial of text4baby at Madigan Army Medical Center. Female military health beneficiaries who met inclusion criteria were eligible for the study. Participants provided consent, completed a baseline questionnaire, and then were randomized to enroll in text4baby or not. They were followed up at 3 time points thereafter through delivery of their baby. Generalized estimating equation models were used to evaluate outcomes. We examined treatment effects and the effects of higher doses of text4baby messages on outcomes.

**Results:**

We report descriptive statistics including dosage of text messages delivered. The main finding was a significant effect of high exposure to text4baby on self-reported alcohol consumption postpartum (OR 0.212, 95% CI 0.046-0.973, *P*=.046), as measured by the question “Since you found out about your pregnancy, have you consumed alcoholic beverages?” The text4baby participants also reported lower quantities of alcohol consumed postpartum.

**Conclusions:**

Studies of text4baby have helped to build the mHealth evidence base. The effects of text4baby offer lessons for future scalable mHealth programs and suggest the need to study dose-response effects of these interventions.

## Introduction

Mobile health (mHealth), the use of mobile phones as a tool for health care treatment and public health behavior change, is a rapidly expanding field that has significant promise to improve public health and increase the effectiveness of disease prevention and health promotion programs [1-3]. Mobile phones are poised to be powerful tools to promote health in a number of domains and settings worldwide [4]. In particular, mHealth programs have proven effective in drug adherence, as patient reminder systems, and in some areas of areas of chronic disease management, prevention, and control [5,6]. Researchers from multiple disciplines, including population health, social sciences, computing, and engineering sciences are engaged in mHealth [1], and major government research sponsors including the National Institutes of Health and major private sponsors such as the Bill and Melinda Gates Foundation have initiatives in these areas [7-8].

Some of the best mHealth evidence comes from smoking cessation studies [9,10]. Free and colleagues [11] systematically reviewed the evidence on mHealth interventions and found that antiretroviral treatment (ART) and smoking cessation interventions had sufficient evidence of effectiveness to be considered for inclusion in health care services. The authors noted that the ART and smoking cessation studies exhibited no evidence of bias and had significant effects on reduced viral load (ART) and biochemically validated smoking cessation [11]. Abroms and colleagues designed and evaluated the text2quit intervention, which delivers text messages using a tailored feedback approach to promote smoking cessation, and found that 11.1% of former smokers who participated in the intervention remained abstinent after 6 months compared to 5.0% among the comparison group [12]. Whitaker and colleagues [13] specifically reviewed mobile phone-based cessation interventions and found them to be effective in long-term quitting outcomes.

Although the evidence from mobile phone studies is less conclusive in these areas, preliminary research has shown promise in delivering healthy eating and active living (HEAL) interventions. Patrick and colleagues [14] found that, compared to control, short message service (SMS) text messaging and multimedia message service (MMS) participants achieved 1.97 kg greater weight loss. A follow-up study among overweight men also found higher weight loss using Internet and mobile phone [15]. Hurling and colleagues [16] found that an Internet and mHealth intervention among overweight adults that included reminders produced more than 2 hours more physical activity (PA) per week compared to adults with no access. Joo and colleagues [17] found that weekly text messages about diet and PA behavior promoted weight loss. Additionally, there have been studies that used combinations of mobile phone and other new technologies for weight control and found positive effects [18]. A study of postpartum women employed a team approach to encourage women to use a Facebook app to promote PA [19].

Additionally, there is growing evidence that mHealth programs are effective in promoting diabetes self-management [20], as treatment adherence tools, and as reminder systems for health behavior and treatment [11]. Although the evidence for use of mobile phones beyond these areas is still emerging, the overall trend is that they can be effective tools for health promotion, disease prevention, and as adjunct treatment tools.

Interventions using mobile phones are growing worldwide, and some are achieving significant scale and population-level reach. As reported by the United Nations Foundation, Project Masiluleke in South Africa reaches 1 million people each day via mobile phone with human immunodeficiency virus (HIV) prevention, testing, and treatment information [21]. In Mozambique, mobile phones are used to link inventory, distributors, and marketers of socially marketed products, such as condoms and mosquito bed nets, to reach low income consumers [22].

Although the literature from rigorous studies of smoking cessation, treatment adherence, and some other chronic disease prevention and management studies show that mHealth programs can promote behavior change, more randomized controlled trials (RCTs) are needed [11]. There is also a need for more studies on what specific features and approaches in mHealth programs are most effective, including studies on the optimal timing of messaging, dose of exposure, and value of interactive feature [23].

**Figure 1:**
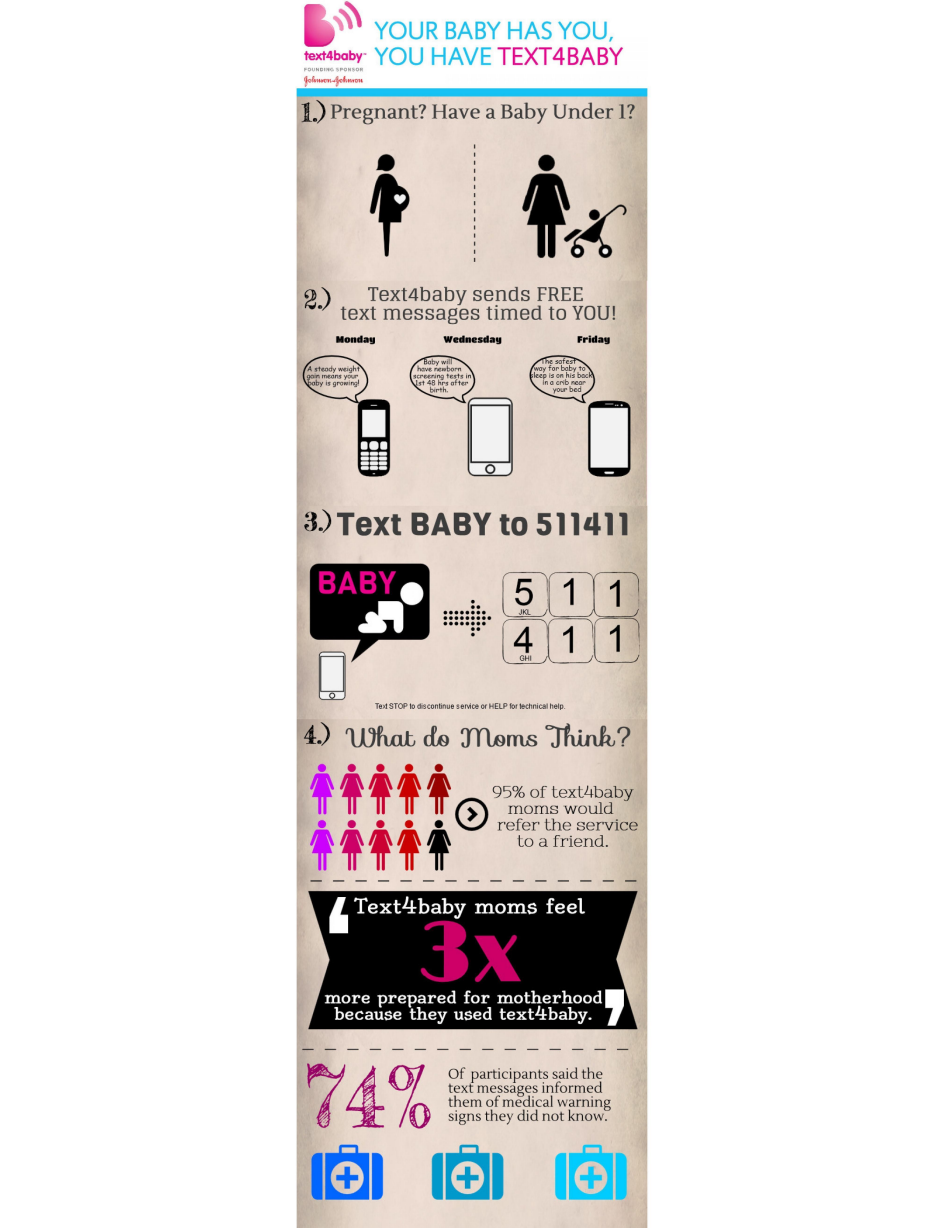
Screenshot of You Have text4baby.

The text4baby program is an example of scaled mHealth for behavior change [24](see Figure 1). The service launched in February 2010 and delivers text messages to pregnant women and new mothers. Since inception, it has enrolled more than 700,000 participants and is one of the largest public health text messaging programs in the United States. It consists of a library of prenatal and postpartum text messages delivered on a schedule timed to the baby’s due date or birthdate [25]. It represents one of the largest mHealth text-based programs developed to date. Recent studies of the text4baby program have found that it changes attitudes and beliefs, but effects on behavior have not previously been established [23,26].

The text4baby program also offers an example of using behavioral theory to the design mHealth interventions [1]. The program applies and adapts Social Cognitive Theory, the Health Belief Model, and Diffusion of Innovation theory [27-29]. The program is branded as a “trusted friend and advisor” and promotes a broad range of maternal and child health behaviors [30]. Future mHealth interventions may build on these lessons to engage audiences in health programs through multiple digital channels [31,32].

The US military has made major investments in mHealth programs to promote health and improve treatment among enlisted personnel and their families [32]. Female service members and spouses of active duty soldiers are a group exposed to multiple stressors that may increase health risks during pregnancy [33-35]. These soldiers and family members could benefit significantly from mHealth programs such as text4baby because it is a free resource and is highly portable, permitting families to receive these messages anywhere in the world where text messages can be received.

The present study evaluates the final outcomes of text4baby from a randomized trial of pregnant female soldiers and family members [23]. The study tested 2 primary hypotheses: (1) text4baby group participants would demonstrate increased health-promoting behaviors and decreased risk behaviors compared to control at postpartum follow-up and (2) text4baby participants would demonstrate a dose-response effect of receiving the text messages in which higher doses of text messages would produce greater behavioral outcomes.

## Methods

### Design and Measures

As previously reported [23], the investigators conducted an RCT of text4baby prenatal messages at Madigan Army Medical Center (Madigan), a large tertiary-care Army Medical Center in Tacoma, Washington. Female military health care beneficiaries aged 18-45 years who first presented for prenatal care at Madigan prior to 14 weeks gestation were eligible for the study. Participants were also required to have a working cell phone, and speak and read English. Following medical consultation, the health care provider asked if the patient would be willing to participate in the research study and those who agreed underwent informed consent and then were offered the opportunity to complete a baseline survey on a secure computer in a private setting in the clinic. Follow-up data collection was conducted remotely primarily through a secure survey website [23].

After baseline survey completion, participants were assigned to the text4baby (plus usual care) or to control (usual care only) groups. We used an algorithm to generate randomized individual assignments to condition for each participant. After baseline, participants in both groups were surveyed again after 4 weeks, at 28 weeks of gestation, and at the time of first postpartum medical appointment. Data collection started in December 2011 and follow-up ended in January 2013. This study reports on analysis of the full dataset from baseline to postpartum follow-up. Detailed data collection procedures have been published elsewhere [23].

The survey instrument included 24 validated items on participant attitudes, beliefs, and behaviors related to the text messages contained in text4baby, which were repeated in the 4 surveys in this study as described in detail elsewhere [23]. Participants took an average of 12 minutes to complete the questionnaire. Follow-up participants answered additional questions on recall, reactions, and receptivity to the texts. Investigators separately obtained available demographic information from each participant’s medical record. The study was approved as minimal risk research by the Madigan Institutional Review Board (IRB) on July 26, 2011. The George Washington University approved this study under an institutional agreement with the Department of Defense (DOD) Human Research Protection Program on August 1, 2011.

### Intervention

Participants assigned to the control condition were excused immediately after completing the baseline survey. The on-site study coordinator assisted treatment participants in enrolling in text4baby by texting “DODBABY” to a designated SMS short code that tagged them as participants in the study. Voxiva, the information technology firm that delivers the text4baby messages and maintains data provided by participants on enrollment, maintained records to identify participants as members of the Madigan study. More information on Voxiva and text4baby has been published elsewhere [24]. Only text4baby participants who were enrolled in the Madigan study were counted in our treatment group. We monitored the control group to ensure that none of these participants separately enrolled for text4baby (none did).

Voxiva collected data on all text4baby participants through enrollment and follow-up service contacts [36]. As noted, we received information on enrollment and service end dates, and from those data determined the number of texts delivered to participants as part of the Madigan study. This was covered under a data use agreement (DUA) between the Department of Defense, National Healthy Mothers, Healthy Babies (HMHB) Coalition, Voxiva, and The George Washington University. HMHB and Voxiva, joint owners of the text4baby service, did not have access to any data collected through the study or any patient information stored at Madigan. Participants in the study received 3 text messages per week throughout their enrollment, which were tailored to the date of enrollment and gestational age. Slight modifications to the standard text4baby messages were made due to specific health care resources (eg, toll-free numbers for prenatal health information) available to military women [23,25]. The standard text4baby message libraries are summarized on the text4baby website [37].

If a fetal loss occurred at any time during the pregnancy, patients were disenrolled from text4baby and appropriate perinatal grief counseling was offered to the patient and her partner. Patients were also provided the option to disenroll from text4baby by texting “STOP” from their mobile phones if they no longer wanted to participate in the program.

### Sampling

As detailed elsewhere, the sampling frame consisted of all female military health care beneficiaries first presenting for initial prenatal care at Madigan. We drew a random sample of all women meeting criteria at the Madigan Obstetrics and Gynecology Clinic between December 2011 and January 2013. Recruitment took place over this time period until the targeted sample was reached. Follow-up data collection began in January 2012 and was completed by September 2013. Previous interventions to promote reproductive health care utilization among low- and middle-income women suggested an approximate 12% effect (intervention vs control) of such programs after a 12-month time period [38]. Power analysis estimated the required sample to be 996 participants in total assuming a 10% attrition rate at postpartum follow-up.

### Data Collection Procedures

Investigators held an introductory meeting and training session on the study protocol with clinical staff at Madigan in November 2011. Surveys were self-administered and baseline surveys were completed usually in clinic, with a small number completed remotely online. Follow-up surveys were primarily completed remotely online. We used several methods to address noncontact and noncooperation, including (1) text messages, (2) a local phone number for participants to call the investigators or a clinic nurse with questions, and (3) assurances of confidentiality. Reminder texts were sent 1 week before each follow-up survey prompting online or in-clinic survey completion. Participants were considered to have quit the study if they were unreachable after 7 recontact attempts, and no further follow-up attempts were made.

### Data Analysis

Stata version 12 (StataCorp LP, College Station, TX, USA) was used in all analyses. Analyses were conducted from March 24 to June 13, 2014. Descriptive statistics including means, percentages, and standard deviations were calculated for all outcomes and demographic variables. Crosstabs of these same variables by study condition and survey time points were also calculated. Also, intervention exposure, as measured by estimated number of antepartum messages delivered and number of antepartum days enrolled in the messaging campaign, were assessed for all study participants in the intervention group. Based on the distribution of messages, investigators examined estimated dosage levels, dichotomized on whether a participant received lower or higher than median message exposure to messaging. Exposure was measured by an estimated count of total messages delivered to participants based on service enrollment and end dates validated by the Voxiva text4baby service database, which were linked to individuals and provided to the investigators under the DUA.

Generalized estimating equations (GEE) logistic regression was used to construct separate models for each of the attitudes, beliefs, and behavioral outcomes over the 3 follow-up periods, estimating population-attributable effects for treatment vs control groups, and high (more than the median message exposure of 224 days) vs lower intervention exposure. Investigators estimated the odds of change over time in response to each of the behavioral outcome variables as a function of text4baby text message exposure through use of an interaction term including program exposure and progression to follow-up measurement.

In addition to an unadjusted model, which strictly looked at the effect of the intervention on those who completed baseline and at least 1 follow-up interview (n=459), a second adjusted model included several maternal covariates: age quintile, parity, marital status, and race. For missing data and attrition of participants, a *t* test was used to compare covariates, including sociodemographic and other variables used in the regressions, between cases with and without missing data to verify whether or not data were missing completely at random. It was determined that both maternal race and marital status (both missing for 215/943, 22.7% of baseline participants) were potentially variables that were differentially missing for women of certain racial and marital statuses. Therefore, a multiple imputation model was constructed to account for missing race and marital status through use of a logit function with parity, age, and treatment status as predictors of both race and marital status.

A confirmatory factor analysis of related knowledge, attitudes, and beliefs (KABs) was also conducted to examine correlation in agreement and changes in agreement of beliefs. A factor loading criteria of 0.4 was used to determine whether to retain individual items in a single principal factor. Following widely accepted practice, Cronbach alpha of .6 was used to confirm interitem agreement for use of the factor in descriptive and regression analyses [39]. We found that 6 of the 9 KAB items formed a single factor. However, GEE models using the KAB factor did not show significant results and have not included those data.

## Results

**Figure 2:**
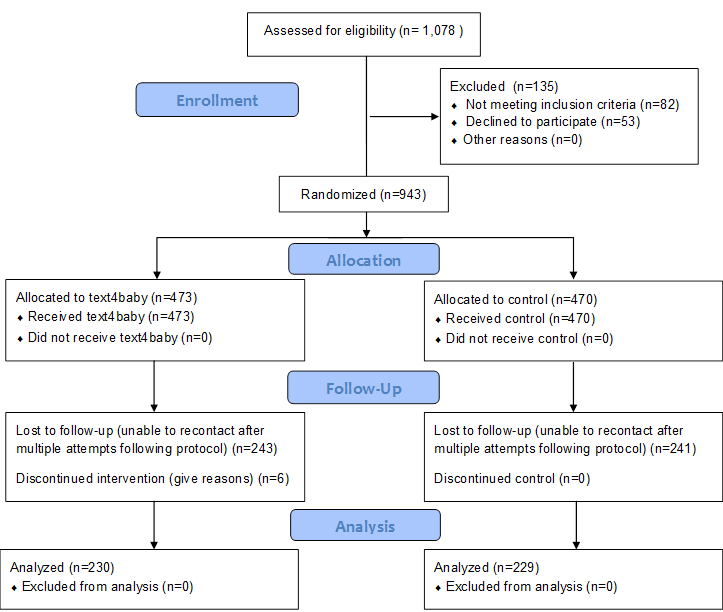
Madigan text4baby CONSORT flow diagram.

Of 1078 women who presented for care during the study period, 996 met criteria and were asked to participate (92.39%). Of these, 94.7% (943/996) completed a baseline survey. Among the baseline participants, 48.7% (459/943) completed a 4-week follow-up survey and 24.5% (231/943) completed a postpartum follow-up. A total of 6 participants discontinued text4baby during the follow-up period. Figure 2 depicts the CONSORT flow diagram for the study.

Table 1 provides the baseline sample characteristics. Overall, the sample was predominantly white (69.6%, 656/943), with a mean age of 26.5 (SD 74.4) years. Most (63.1%, 595/943) reported currently attending school or working outside of the home. A total of 70.3% (663/943) of the participants reported being married. Nearly half of the participants (47.8%, 451/943) reported having had a prior live birth. The vast majority of participants were enlisted service members or a dependent family member of an enlisted service member (86.8%, 819/943) and all but 4 of the remainder were commissioned or warrant officers (14.2%, 120/943).

**Table 1:** Baseline sample descriptive statistics (N=943).

Variables		Participants
Age (years), mean (SD)		943 (26.5)
**Age range (years), n (%)**		
	<20	31 (3.3)
	20-34	837 (88.8)
	≥35	75 (7.9)
**Race, n (%)**		
	White	656 (69.6)
	Black	75 (7.9)
	Asian-Pacific Islander	25 (2.6)
	Western Hemisphere Indians	2 (0.2)
	Other/unknown	185 (19.6)
**Ethnicity, n (%)**		
	Filipino	206 (21.8)
	Hispanic	53 (5.6)
	Other Asian/Pacific Islander	19 (2.0)
	Southeast Asian	6 (0.6)
	Other/unknown/non-Hispanic	659 (69.9)
**Marital status, n (%)**		
	Single/never married	72 (7.6)
	Married	663 (70.3)
	Separated/divorced/widowed	7 (0.7)
	Unknown/null	201 (21.3)
**Sponsor rank, n (%)**		
	Enlisted	819 (86.8)
	Commissioned officers	107 (11.3)
	Warrant officers	13 (1.4)
	Other	4 (0.4)
**Parity, n (%)**		
	No	492 (52.2)
	Yes	451 (47.8)
Prepregnancy body mass index (BMI), mean (SD)		327 (27.2)
**BMI category, n (%)**		
	Underweight	7 (0.7)
	Normal	154 (16.4)
	Overweight	97 (10.3)
	Obese	63 (6.7)
Ever participated in WIC program, n (%)		320 (33.9)
Currently in school or working outside the home, n (%)		595 (63.1)
Ever gone online to search for prenatal care information, n (%)		711 (75.4)

Equivalence of means at baseline was tested by comparing the baseline treatment and control condition samples. The comparison revealed a larger, statistically significant percent reporting smoking in the last 30 days: 15.3% (95% CI 12.08-18.58) in the control vs 9.6% (95% CI 6.95-12.32, *P=*.048) in the treatment group, respectively. There was also a larger, statistically significant percentage who reported consuming 3 or more vegetables per day in the control vs treatment group: 37.8% (95% CI 33.44-42.19) in control vs 30.0% (95% CI 25.81-34.15, *P*=.046) in the treatment group, respectively.

As noted, we obtained data on the number and timing of text messages delivered to the text4baby participants. Three text messages were delivered per week at random times during waking hours on weekdays. We identified the week of pregnancy in which participants enrolled using the assumption of a 40-week gestation period and subtracting the total number of days between the participant’s date of enrollment and due date recorded by the study site. Using this calculation, we identified the number of messages delivered to each participant during the study period. This calculated variable was used for the GEE dose-response models. Table 2 displays a summary of the mean messages delivered to participants based on the calculated variable, standard deviation, and range of estimated total text messages delivered.

**Table 2:** Summary of pregnancy text4baby text messages delivered (n=192).

Text message delivery variables	Mean (SD)	Range
Total number of messages sent	61.3 (43.2)	10-151
Week of pregnancy when enrolled	8.1 (1.9)	4-14
Weeks in pregnancy protocol	12.6 (10.9)	0-33.7

Table 3 presents results of unadjusted and adjusted versions of the first set of GEE models. In these models, we examined the effects of text4baby participation (treatment condition) on postpartum KAB and behavioral outcomes targeted by the text messages. No significant effects of text4baby were observed in these models.

**Table 3:** Adjusted and unadjusted GEE models comparing treatment (text4baby group) and control (no text4baby enrollment).

Questionnaire items		Treatment vs control			
		Unadjusted		Adjusted^a^	
		OR (95% CI)	*P*	OR (95% CI)	*P*
**KAB items**					
	Eating 5 or more fruits and vegetables per day is important to the health of my developing baby	1.781 (0.409, 7.760)	.44	2.261 (0.400, 12.787)	.36
	Taking a prenatal vitamin is important to the health of my developing baby	2.931 (0.463, 18.551)	.25	1.512 (0.162, 14.092)	.72
	I am prepared to be a new mother	0.923 (0.563, 1.514)	.75	0.976 (0.495, 1.924)	.94
	If I visit my health care provider on a regular basis, I will be a healthy new mother	1.016 (0.666, 1.549)	.94	0.759 (0.461, 1.251)	.28
	If I visit my health care provider on a regular basis, my baby will be healthy	1.188 (0.771, 1.829)	.43	0.949 (0.595, 1.513)	.83
	Smoking will harm the health of my developing baby	0.880 (0.583, 1.328)	.54	0.753 (0.473, 1.198)	.23
	Secondhand smoke will not harm the health of my developing baby (reverse coded)	1.189 (0.944, 1.497)	.14	0.978 (0.715, 1.336)	.89
	Drinking alcohol will harm the health of my developing baby	1.018 (0.619, 1.675)	.94	1.253 (0.717, 2.188)	.43
	Taking prenatal vitamins will improve the health of my developing baby	0.943 (0.722, 1.233)	.67	0.709 (0.488, 1.030)	.07
**Behaviors**					
	Since you found out about your pregnancy, have you consumed alcoholic beverages?	0.699 (0.293, 1.665)	.43	2.310 (0.491, 10.874)	.29
	In the last 30 days, did you smoke?	0.927 (0.533, 1.615)	.79	0.938 (0.318, 2.770)	.91
	Ate 3 or more servings of fruit a day	1.160 (0.924, 1.456)	.20	1.107 (0.835, 1.468)	.48
	Ate 3 or more servings of vegetables a day	1.065 (0.846, 1.339)	.59	1.042 (0.755, 1.438)	.80
	Have you ever gone online to search for prenatal care information?	0.853 (0.584, 1.246)	.41	0.822 (0.476, 1.420)	.48

^a^ Adjusted for age, parity, imputed marital status, and race.

Table 4 presents results of unadjusted and adjusted versions of the second set of GEE models. In these models, we examined the effects of high vs low dosage of text4baby, as measured by a median split variable in which the top 50% of the distribution of text message exposure among text4baby intervention participants was compared to the bottom half among that same group. In these models, we found a significant effect of text4baby on self-reported alcohol consumption postpartum (OR 0.212, 95% CI 0.046-0.973, *P*=.046), as measured by the question “Since you found out about your pregnancy, have you consumed alcoholic beverages?” The lower OR indicates lower odds of consuming alcoholic beverages at postpartum follow-up.

**Table 4:** Comparison of high and low exposure to text4baby messages: adjusted and unadjusted GEE models.

Questionnaire items	High vs low exposure			
	Unadjusted		Adjusted^a^	
	OR (95% CI)	*P*	OR (95% CI)	*P*
Eating 5 or more fruits and vegetables per day is important to the health of my developing baby	0.744 (0.138, 4.003)	.73	2.511 (0.158, 39.867)	.51
Taking a prenatal vitamin is important to the health of my developing baby	0.760 (0.115, 5.000)	.77	4.216 (0.201, 88.417)	.35
I am prepared to be a new mother	1.471 (0.788, 2.746)	.22	1.609 (0.607, 4.268)	.34
If I visit my health care provider on a regular basis, I will be a healthy new mother	1.033 (0.607, 1.759)	.90	1.288 (0.585, 2.834)	.53
If I visit my health care provider on a regular basis, my baby will be healthy	0.783 (0.468, 1.313)	.35	1.328 (0.737, 2.393)	.34
Smoking will harm the health of my developing baby	1.139 (0.717, 1.810)	.58	1.236 (0.651, 2.350)	.52
Secondhand smoke will not harm the health of my developing baby (reverse coded*)	0.892 (0.668, 1.193)	.44	0.947 (0.631, 1.423)	.79
Drinking alcohol will harm the health of my developing baby	1.088 (0.655, 1.808)	.74	0.803 (0.451, 1.431)	.45
Taking prenatal vitamins will improve the health of my developing baby	1.090 (0.782, 1.520)	.61	1.378 (0.861, 2.204)	.18
Behaviors				
Since you found out about your pregnancy, have you consumed alcoholic beverages?	1.344 (0.473, 3.820)	.58	0.212 (0.046, 0.973)	.046
In the last 30 days, did you smoke?	0.938 (0.490, 1.794)	.85	1.271 (0.406, 3.980)	.68
Ate 3 or more servings of fruit a day	0.852 (0.647, 1.122)	.25	0.908 (0.649, 1.271)	.57
Ate 3 or more servings of vegetables a day	0.889 (0.679, 1.164)	.39	0.969 (0.673, 1.394)	.86
Have you ever gone online to search for prenatal care information?	0.992 (0.661, 1.488)	.97	0.893 (0.519, 1.536)	.68

^a^ Adjusted for age (quintile), parity, imputed marital status, and race.

Additionally, if participants answered yes to having consumed alcoholic beverages, we asked them “How many drinks do you normally drink per day.” At baseline, 97.3% (918/943) of all participants reported zero drinks per day and this rate remained fairly constant at 97.6% (448/459) at the first follow-up, with no differences between study conditions. At the postpartum follow-up, the overall rate declined to 55.4% (128/231). However, of the 128 respondents who indicated zero drinks per day, only 51 (40%) of them were in the control group and 77 (60%) in text4baby.

## Discussion

The text4baby program is significant in that it represents one of the largest worldwide mHealth programs to date, with more than 800,000 enrollees from inception in February 2010 through November 2014 [40]. To date, there have been very few large-scale mHealth programs shown to be effective. The future of mHealth will include going beyond small-scale pilots and trials and reaching population-level effects through large-scale implementation.

This study is significant for at least 3 reasons. First, it provides a randomized trial of text4baby with a large sample over the full course of the prenatal texting module. The Madigan study was ambitious in that it followed a baseline sample of nearly 1000 women for a total study period of nearly 2 years. Second, it provides one of the most comprehensive studies to date evaluating the effect of mHealth on the health of pregnant military female soldiers and family members. As noted, military women’s health is an understudied topic and the multiple stressors that they face compared to civilian women suggest that it deserves greater attention from mHealth and other health studies [34,41]. Third, the study demonstrates a behavioral effect of text4baby among high-exposure users. Previous studies demonstrated short-term effects on KAB among participants, including the initial outcomes of the Madigan study [23,26]. However, until these results, no behavioral effects had been observed. We found that among the subgroup of high-exposure participants, text4baby had a positive effect on reducing alcohol use behavior of pregnant and postpartum women.

Specifically, we disconfirmed our first hypothesis that there was no direct treatment effect of text4baby. The GEE models to estimate treatment effects on measured outcomes did not demonstrate any significant postpartum text4baby participation effects on health-promoting behaviors. However, we confirmed our second hypothesis that there was a dose-response effect of text4baby, with higher levels of text message exposure predicting lower self-reported alcohol consumption. This was an important focus of text4baby messages, including both recommendations not to drink and also warnings about the health risks of fetal alcohol syndrome for unborn babies. Our results show that dosage was a predictor of lower alcohol consumption response. Although self-reported alcohol consumption would be expected to be lower after pregnancy given social cues and available information about health risks for all participants, the dose-response effect among text4baby participants was pronounced, as high-dose participants were more than twice as likely to abstain from drinking compared to low-dose participants. The brief, text-based intervention format of text4baby is consistent with other brief prenatal alcohol interventions found to be effective in recent studies [42].

Moreover, descriptive analysis showed that the quantity of drinking was lower among text4baby participants postpartum. In total, there were 6 messages related to risks of alcohol consumption, spread over the intervention period, among the pregnancy messages delivered. Although this is not a large number of total messages, it is worth noting that they reinforce information women are already receiving from other sources regarding avoiding alcohol use. These data suggest that dosage of texts received regarding risks of alcohol use, which included messages regarding risks due to alcohol while breastfeeding, had a cumulative effective.

There are several implications of the Madigan study and text4baby for future mHealth interventions and research. First, text4baby is a broad, relatively “low-touch” intervention. The program addresses a wide range of health topics, as compared to other studies that focused on 1 or a few closely related health behaviors. It does not include substantial participant investment of time and effort, or interactivity. However, initial short-term findings demonstrated multiple KAB effects [23], and this study demonstrated a behavioral effect on alcohol use. Broad, low-touch interventions can be effective and given low participant burden should be considered as scalable program options.

There is a need to understand optimal levels of dosage and other factors that affect mHealth intervention outcomes. It is important to recognize that many individuals who use text messages receive large numbers of texts per day. We did not have measures of total texts or potentially competing messages received by participants in this study, and those are important topics for future research.

Delivering high doses of mHealth interventions has implications in terms of cost, participant burden, and potential “wear out” effects (ie, overexposure). Thus, identifying optimal mHealth dosages could have potential major benefits for future programs in terms of cost effectiveness and outcomes. Although this study does not indicate an optimal dose, it suggests the need to understand dosage thresholds and delivery methods.

Future research should include more discrete and refined dosage and other optimization studies. For example, studies have examined point-of-decision prompts to increase exercise and nutrition [43], and use of mobile technologies for health interventions [11,44], but no study has combined both. Text messaging interventions can do much more than simply deliver text reminders—they can deliver right into the hands of highly targeted population the public service announcements that in years past would have appeared in mass media [45]. Future interventions can tailor text message and other mHealth message content (eg, through social media or apps) both to a specific target audience and to an optimal time for delivery. By getting messages to a specific population group when they are at risk of engaging in unhealthy behavior (eg, teens watching TV, being exposed to junk food advertising, being sedentary and snacking at times such as after school or on weekends), interventions can influence them at the optimal time [46,47].

There are 2 important limitations of this study. First, we had a low follow-up rate and, thus, used imputation techniques to support the analysis. As a result, the study may be underpowered. Despite this fact, the overall direction of results was consistent with previous text4baby studies and additionally we found a behavioral effect. Much of the attrition was attributable to redeployments and, thus, lack of access to participants at the Madigan clinic. Second, although we observed dosage effects, we are only able to conclude that higher levels of text messages had an effect on alcohol use. This leaves the important question of exact dosage and timing of delivery requirements for future studies.

Studies of text4baby have helped establish and expand the mHealth evidence base. The demonstrated KAB and behavioral effects of this broad, low-touch program offers lessons for future scalable mHealth efforts. The dose-response effects observed here suggest the need to study methods to evaluate exposure and achieve optimal dosage effects in future research. Program dosage and optimization research should be included to address other features of the mobile phone, such as ubiquity, constant use, and potential to act as a point of decision prompt.

## Abbreviations

ART: antiretroviral treatment
BMI: body mass index
DOD: Department of Defense
DUA: data use agreement
GEE: generalized estimating equation
HEAL: healthy eating and active living
HIV: human immunodeficiency virus
HMHB: Healthy Mothers, Healthy Babies Coalition
IRB: Institutional Review Board
MMS: multimedia message service
PA: physical activity
RCT: randomized controlled trials
SMS: short message service
